# Motion Estimation by Hybrid Optical Flow Technology for UAV Landing in an Unvisited Area

**DOI:** 10.3390/s19061380

**Published:** 2019-03-20

**Authors:** Hsiu-Wen Cheng, Tsung-Lin Chen, Chung-Hao Tien

**Affiliations:** 1 Department of Mechanical Engineering, National Chiao Tung University, Hsinchu 30010, Taiwan; zintwen@gmail.com (H.-W.C.); tsunglin@mail.nctu.edu.tw (T.-L.C.); 2 Department of Photonics, National Chiao Tung University, Hsinchu 30010, Taiwan

**Keywords:** optical flow, unmanned aerial vehicle (UAV), vision-based motion estimation

## Abstract

The capability of landing on previously unvisited areas is a fundamental challenge for an unmanned aerial vehicle (UAV). In this paper, we developed a vision-based motion estimation as an aid to improve landing performance. As an alternative to the common scenarios accompanying by external infrastructures or well-defined marker, the proposed hybrid framework can successfully land on a new area without any prior information about guiding marks. The implementation was based on the optical flow technique associated with a multi-scale strategy to overcome the decreasing field-of-view during the UAV descending. Compared with a commercial Global Positioning System (GPS) through a sequence of flight trials, the vision-aided scheme can effectively minimize the possible sensing error, thus, leading to a more accurate result. Moreover, this work has potential to integrate the fast-growing image learning process and yields more practical versatility for UAV applications in the future.

## 1. Introduction

Landing is the most critical and dangerous phase of a flight due to the uncertainty from complex terrains or unpredictable occurrence. Unlike the manned aircraft with pilots capable of responding to various environmental interferences, an Unmanned Aerial Vehicle (UAV) needs to equip a vision system to retrieve related information, especially when a UAV lands on an unvisited area. In this study, we attempt to develop a vision system as an aid for UAV landing purposes. Unlike conventional scenarios structured with external infrastructures such as Differential Global Positioning System (DGPS) and trajectory measurement system, the proposed vision-based scheme provides more versatilities in integrating the fast-growing imaging techniques into aircraft control. 

Vision-based motion estimation for aerial vehicles firstly experimented on a fixed-wing manned aircraft [1]. This work demonstrated that visually estimated altitude and longitudinal position can have a comparable measure to those from the DGPS system. For the rotorcraft applications [2], the vision system has usually been employed to navigate and orient the UAV with a helipad in view. Specific patterns such as a ring pattern [3,4] or fan-shaped marker [5] are marked as the guides to tackle the close range and nighttime detection problem during UAV descent. Accompanying a trained marker model via Convolutional Neural Network (CNN), the tracking range can be extended to a 50m-height until the UAV successfully lands on the ground [6]. In addition to the static platform, numerous works were addressed on a moving target. Due to the necessity of collaboration between a UAV and a moving platform, the velocity information of the moving platform plays a key role in the landing process. Two schemes either optimizing the marker detection rate [7] or exploiting the moving target’s dynamic model were developed [8,9]. In summary, the aforementioned works either focused on static or dynamic targets, the main purpose of which was to deal with the missing tracked features when the field-of-view was subject to the flight height. On the other hand, for landing in an unvisited environment, it is unlikely to place a structured landing marker in advance, and the identification of a safe landing area becomes another considered issue. 

Accordingly, some studies have aimed to assess the landing risk by evaluating the planarity and slope of the terrain or construct a partial map for navigation purposes [10,11,12]. To learn the properties of a landing surface, a database of known textures is used to train the supervised terrain classifier to identify suitable landing areas [13]. Recently, a neural network policy has been trained to navigate the UAV by imitating pilot behavior generated from wheeled manned vehicles [14]. Despite the fact that introducing vision-based functions can improve the landing capability, most studies addressed the motion estimation subject to a well-defined landing target. To our knowledge, in an unsurveyed environment, the recognition capability may not detect the target that meets the feature criteria preset in the algorithm, and the environmental survey aimed at the landing site selection rather than the motion estimation during landing. 

The optical flow techniques, correlating two consecutive image frames, were proven to be effective in motion detection [15,16]. Two major approaches subject to the feature dimension can be divided into sparse [17,18] and dense optical flow [19,20,21]. Driven by different applications, the sparse optical flow attempts to find a few useful features from images, whereas the dense optical flow needs working on every pixel to find the highest fidelity of the images. When we consider landing on an unvisited area, how to compromise both advantages in terms of computational efficiency and accuracy becomes the aim of this paper. 

The Gunnar–Farnebäck Algorithm (GFA) [22] is a common dense optical flow scenario. In order to save the computation effort, some studies would transform the image into the Fourier domain and extract the corresponding global features by means of phase correlation accordingly [23,24,25,26,27]. In this study, we applied GFA for velocity estimation since the algorithm can precisely find the translated vectors amid two consecutive images subject to small motion requirement [28,29]. On the other hand, the Phase Correlation (PC) method allows more margin against the image mismatch [30,31], which is suitable for the relative position estimation. Furthermore, since the PC method was vulnerable to scale variation during UAV descent, we obliged to modify the PC method by introducing a multi-scale strategy to overcome this issue.

In this paper, we proposed a novel scheme that compromises both the advantages of sparse and dense optical flow techniques. We presented a vision-aided landing system developed for rotorcraft UAV to land on a previously unvisited area. The major functions of the proposed system are to track the landing spot with no marker and provide accurate and reliable measures of both position and velocity to replace the existing GPS measurement in a UAV control system. The remainder is organized as follows. In Section 2, we introduced the mathematical model about GFA and PC method, as well as the multi-scale strategy to tackle the field-of-view problem during UAV descending. In Section 3, we validated the hybrid optical flow scheme via landing experiments and compared the results with the current GPS. Finally, a brief summary and discussion are given in Section 4. 

## 2. Vision-Based Motion Estimation by Hybrid Optical Flow

**Figure 1:**
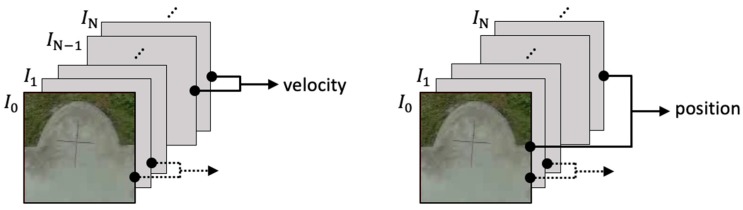
Overview of visual motion estimation: the velocity is obtained by comparing image patterns on two consecutive frames (**left**), and the position is computed by phase correlation method with respect to a reference image (**right**).

In the hybrid optical flow technique, correlating multiple image frames, was proposed to estimate two dynamic measures: velocity and position. As shown in Figure 1, the velocity information is determined by comparing two consecutive frames ($I_{N}-\;I_{N-1}$), whereas the position estimate is determined by the difference between the Nth frame and the reference frame ($I_{N}-\;I_{0}$) to avoid the integral error accumulations. Specifically, once the UAV was commanded to hold its position, the vision system sets the image at that instant as the reference frame ($I_{0}$), and the center of the reference frame as the designated landing spot.

**Figure 2:**
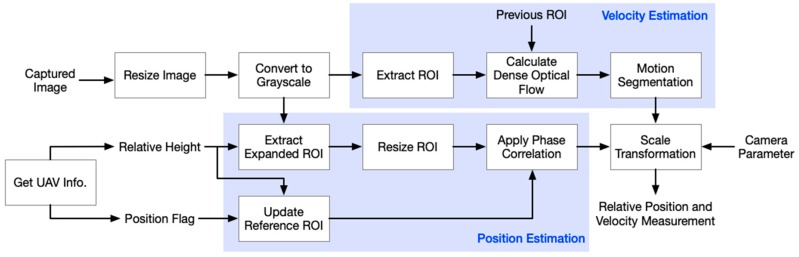
Software flowchart of the vision processing system. The velocity estimation is based on the Grunnar–Farnebäck algorithm, and the position is calculated by the proposed multi-scale phase correlation method.

Figure 2 exhibits the framework of the entire image processing. Firstly, the aviation vision system conducts motion estimation with the data stream from the control system. To save the memory and computational time, the algorithm merely works on the Region-of-Interest (ROI) of the input image stream. We, thus, dynamically adopted two algorithms: GFA and PC to obtain the velocity and position information, respectively. Details about these two methods would be explained in the following content. Finally, both estimated velocity and position information were utilized as the feedback signals to the flight control system.

### 2.1. Grunnar–Farnebäck Optical Flow

While flying a UAV in an unvisited area there is no prior information as the landing reference. Since motion detection is mainly achieved by comparing the image patterns, it is unlikely to retrieve sufficient information as the scene has plain texture. Compared with the conventional global feature-based methods, the Gunnar–Farnebäck optical flow [22] with pixel-wise correspondences between two subsequent images has superior performance [19,20,32]. With each pixel in an ROI, its neighborhood can be characterized by polynomial regression as:

(1) $$f\left(x\right)\sim x^{T}Ax+b^{T}x+c,$$

where x is the coordinate of a pixel in the neighborhood and f(x) is the characteristic value of that pixel. The regression coefficients A, b, c are determined by the weighted-least-square method to fit the grayscale values in the neighborhood, where  A, b, c are in form of matrix, vector and constant, respectively. With two consecutive frames, the translational displacement $d=\left(d_{x},d_{y}\right)$ is small enough, leading the characteristic value of neighborhood are equal:

(2) $$f_{N}\left(x\right)=f_{N-1}\left(x-d\right).$$

Substituting (1) into (2), the displacement d can be obtained:

(3) $$d=-\frac{1}{2}A_{N-1}^{-1}\left(b_{N}-b_{N-1}\right),$$

(4) $$A_{m}=\frac{1}{2}\left(A_{N-1}+A_{N}\right).$$

In practice, $A_{N-1}$ is often replaced by the average of two frames $A_{m}$. Under the assumption that the displacement field is varying slowly, Equation (3) can be generalized as follows to include all the displacement information from the neighborhood:

(5) $$d={\left(\sum^{\;}wA_{m}^{T}A_{m}\right)}^{-1}\sum^{\;}wA_{m}^{T}∆b_{m},$$

(6) $$∆b_{m}=\frac{1}{2}\left(b_{N-1}-b_{N}\right),$$

where w denotes the weight function for each pixel in the neighborhood. 

**Figure 3:**
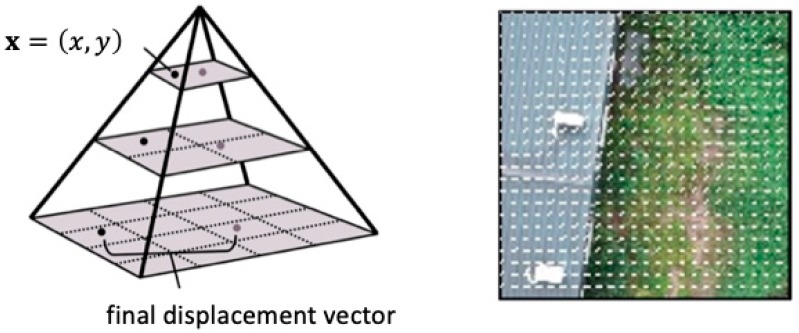
An image pyramid with three levels of pixel resolution. The top level has the lowest pixel resolution while the bottom level has the highest pixel resolution. The iterative Gunnar–Farnebäck algorithm is used to estimate the optical flow from top to bottom. An example of the estimated flow field is shown on the right.

To speed up the computational time, the above algorithm can be modified by the iterative scheme with a prior estimate, whereas a coarse to fine image pyramid was built. As the illustration in Figure 3 (left), the top to bottom represents the increment of pixel resolution. We first computed the displacement vector via the pixels on the top level and then used these values as the prior estimates for computing the displacement vector on the second level. The process was repeated until completing the computation for each pixel on the bottom level. Figure 3 (right) shows the distribution of the displacement vector processed by the GFA. Finally, we could segment the area of interest on the basis of the magnitude, take the average value, and obtain the velocity of the object. This approach provides practical advantages of the high sensing bandwidth and moderate sensing accuracy.

### 2.2. Multi-Scale Phase Correlation

For position estimation with long working range, it is no longer adequate to keep track on every pixel in the ROI due to the large displacement. Instead, we employed the PC method to obtain the translational offset between two images based on the Fourier Shift theorem [33,34]. Consider the two images $\left(I_{0},I_{N}\right)$ and their Fourier counterpart $\left(G_{0},G_{N}\right)$, whose cross-power spectrum is given by

(7) $$R_{0N}=\frac{G_{0}G_{N}^{*}}{\left|G_{0}G_{N}^{*}\right|}=e^{j\theta }.$$

The displacement shift was revealed through the phase term θ in the cross-power spectrum. After the Inverse Discrete Fourier Transform (IDFT), a Dirac delta function comes out with a peak shift $d=\left(d_{x},d_{y}\right)$.

(8) $$r\left(x,\;y\right)={ℱ}^{-1}\left\{R_{0N}\right\}=\delta \left(x+d_{x},y+d_{y}\right),$$

whereas

(9) $$\left(d_{x},d_{y}\right)=arg\underset{\left(x,y\right)}{\max}\left\{r\right\}.$$

**Figure 4:**
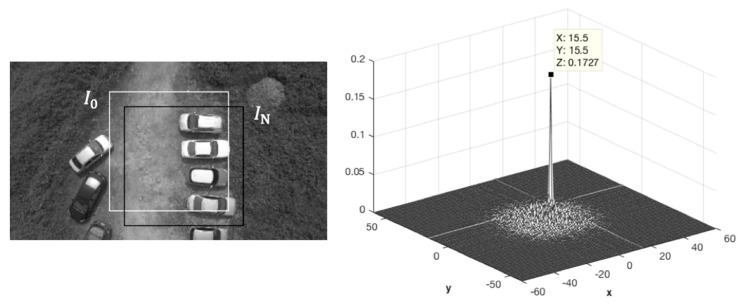
An example of the displacement-finding using the phase correlation method: two source images are cropped from the selected area (**left**), and the resulting peak on the correlation plot (**right**).

Figure 4 shows an example of the displacement-finding using the PC method. The displacement vector subject to $I_{0}$ and $I_{N}$ resulted in a peak shift on the correlation plot. 

**Figure 5:**
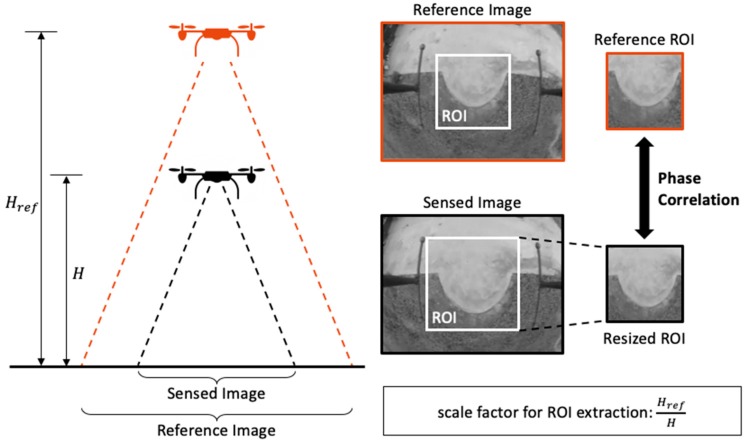
Illustration of the multi-scale phase correlation method. The size of ROI extraction was expanded according to the flight altitude, and, the sensed ROI was resized back to the default size and applied to the phase correlation function afterward.

The typical PC algorithm only allows a small range of scale variance between the two images. However, the image-based motion detection would experience a large-scale difference during the whole landing process. Instead of using spatial correlation methods which are computationally intensive [30], we proposed a multi-scale phase correlation method for relative position estimation during landing. Figure 5 shows the concept of a multi-scale phase correlation method. During the landing process, the decreasing altitude of a UAV would narrow down the field-of-view of the onboard camera. Accordingly, the sensed ROI was enlarged by a factor λ and was resized by the reciprocal of the same factor to keep its scale the same as the reference ROI afterward. The factor λ can be calculated by the height of the UAV:

(10) $$\lambda =\frac{H_{ref}}{H},$$

where $H_{ref}$ and H are the flight altitude of the reference image and the sensed image, respectively. The height of the UAV can be measured by the onboard sensor such as a laser altimeter. 

**Figure 6:**
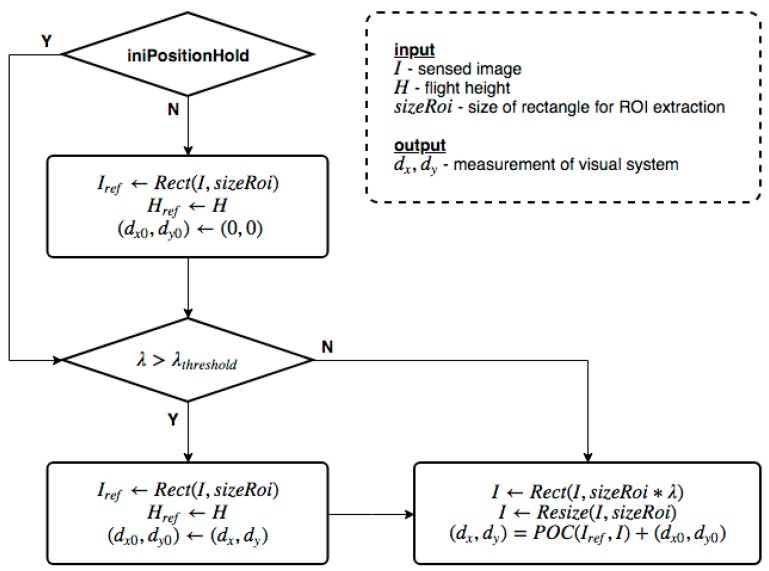
Flowchart of the multi-scale phase correlation. The reference ROI is autonomously updated when λ reaches a threshold value. When crossing different epoch of the reference ROI, the position measurement is carried out with the prior estimate as an initial condition.

Furthermore, as the UAV was descending, an instant updating reference ROI image was essential to make sure that it contains enough area for extracting the ROI out of the subsequently measured images. Figure 6 shows the signal processing flow, where a threshold value $\lambda_{threshold}$ was set to trigger the update of the reference ROI. Within each reference ROI epoch, the currently sensed ROI is resized back to the physical coordinate, and the displacement vector with respect to the reference ROI image is derived accordingly. Finally, the position information is carried on to the next reference ROI epoch.

## 3. Experimental Results

This section reveals the experiment of vision-aided UAV landing in an outdoor environment. After describing the experimental setup, we examined the capability of the hybrid framework for motion estimation. Then, we proceed to present the results of vision-based landing in comparison to GPS-based landing. While the vision function was in effect, the system would simultaneously collect the GPS signal as the benchmark. In terms of velocity, we calculated the mean and the standard deviation of the difference between these two systems. On the other hand, due to the reason that the position measurement from the GPS may have a drifting problem (the error is several meters), we evaluated the position accuracy by template matching method [35] after flight trials. The method is a technique for detecting the location of an image patch (final position) in a source image (initial position). In that way, the landing error can be derived accordingly. All the comparisons were based on the local coordinate of the vision system.

### 3.1. Experimental Setup

**Figure 7:**
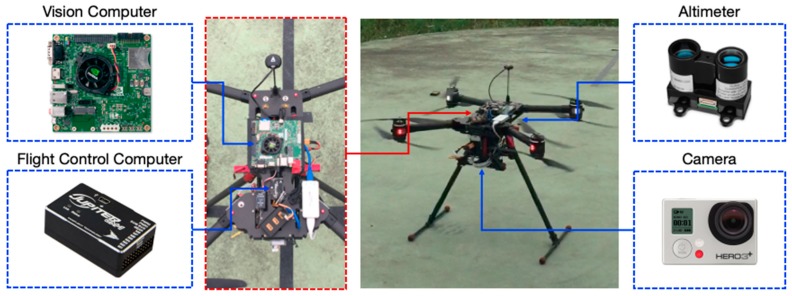
The testbed used in the experiments. In addition to the integrated flight control system, the quadrotor is augmented with a vision processing system and an external camera (30 fps).

The testing UAV as shown in Figure 7, a commercial quadrotor Stellar X1000 from InnoFlight^TM^ (Taoyuan, Taiwan), was pre-installed with a flight control system (Jupiter JM-1 Autopilot), an inertial measurement unit (IMU), a laser altimeter and a GPS module, respectively. For the visual aspect, the image processing system consisted of the NVIDIA^TM^ Jetson TK1 module (Santa Clara, California, USA) and the GoPro^TM^ camera (San Mateo, California, USA). The embedded program executed the algorithms of motion estimation as well as communicated with the flight control computer which runs the proportional–integral–derivative (PID) control scheme.

**Figure 8:**
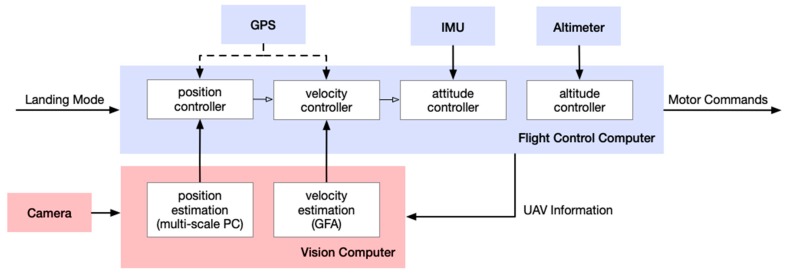
The onboard system architecture of the UAV. According to the landing mode, both position and velocity estimates were obtained from GPS or the vision system respectively, and, the multi-loop feedback scheme was designed for flight control in order to keep the commanded position.

Our goal was to aid the landing with visual feedback and improve the landing accuracy without controller tuning. Figure 8 shows the system architecture and the inter-communication between the controllers. When the visual landing was engaged, both the position and velocity controllers worked on the data acquired by the vision system instead of the GPS. Since the processing time depends on both the image size and the parameters of the algorithms, the acquired image shall be resized to 320 pixels × 180 pixels for the sake of efficiency. After that, the size of 120 pixels × 120 pixels at the center of an image was chosen as the default ROI. With these arrangements, the average processing time was around 36 ms per frame, sufficient for the 100 Hz sampling rate through the control system.

### 3.2. Results and Data Analysis

**Figure 9:**
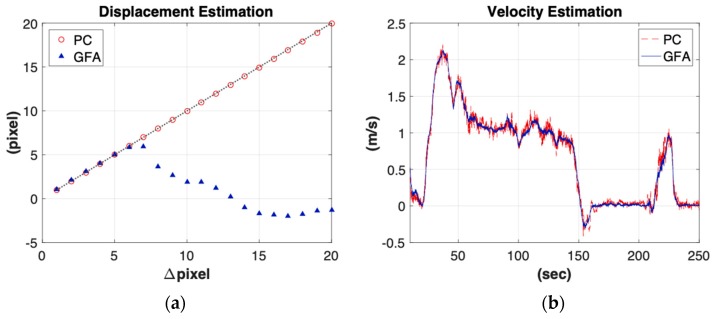
Motion estimation using two vision algorithms: (**a**) For the displacement estimation, the GFA (blue triangle) can detect the shift within 6 pixels whereas the PC method (red circle) can detect the shift up to 20 pixels. (**b**) For the velocity estimation, the results from the PC method (red line) were much noisier than from the GFA optical flow (blue line).

In order to legitimate the GFA and PC algorithm in motion estimation, a ground truth was given in different operational conditions. For position estimation, the difference between the Nth and the reference frame $I_{N}-I_{0}$ (shown in Figure 4) by both GFA and PC methods were individually applied. As shown in Figure 9a, the GFA can successfully track the motion within Δpixel = 6p. As we kept increasing the operational range, the GFA was no longer in agreement with the ground truth. On the other hand, the PC method allows the shifts up to Δpixel = 20p. For velocity estimation shown in Figure 9b, the PC method was vulnerable to the noise due to its inherent sparse property. The GFA, subject to dense optical flow, was more robust at a cost of reducing the operating range. Such experimental results proved the aforementioned statement in Section 2. As a result, in order to compromise the robustness (wide operating range with sparse image features) and accuracy (short operating range with dense image features), the proposed hybrid scheme was individually enabled while velocity or position command were called, respectively. 

### 3.3. Landing Controls using Vision-Based Motion Estimation

To verify the effectiveness of the vision-based landing, we involved the GPS-based landing as the benchmark. The UAV was commanded to hover above a selected landing area, and then steadily descend until the vehicle touched down on the ground. 

**Figure 10:**
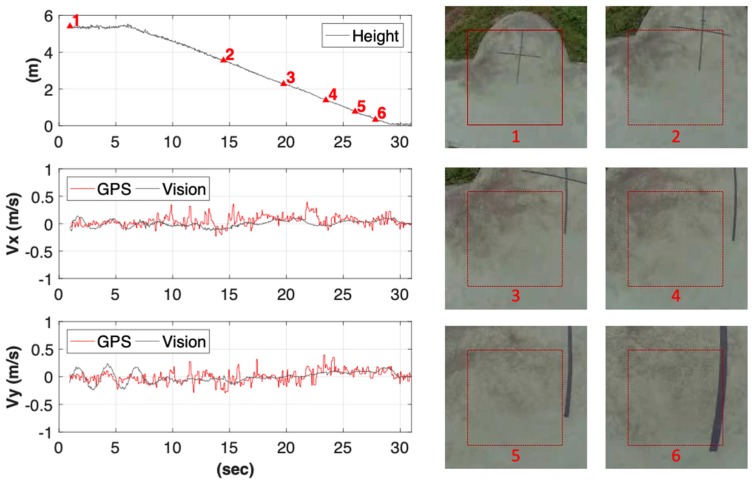
The UAV’s states during visual landing. The reference ROI were updated by the multi-scale phased correlation method based on flight height accordingly, and the resulting velocity estimates of the vision system (black line) followed a similar trend with GPS (red line), with a standard deviation of error about 0.1 m/s in both directions.

**Figure 11:**
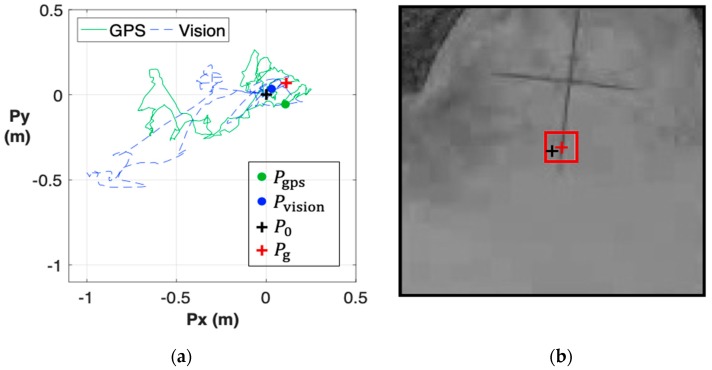
Mode 1 (vision-based landing): (**a**) The in-plane route is obtained from the GPS (green line) and the visual motion estimation system (blue line). The landing process started from the origin (black cross), and was completed at the point marked with a dot; (**b**) The result of template matching shows that the UAV landed approximately 0.1 m away from the designated target.

A video demo for the experiment was shared below: http://tinyurl.com/y656uerp. The flight data along with the captured images were presented in Figure 10 and Figure 11. As shown in Figure 10, while the flight height was decreasing, the reference ROI were updated automatically by the proposed multi-scale phase correlation method, and the updated frequency increased as the vehicle approached the ground to keep scale invariance. It is noted that the black-cross mark on the ground was meant for the analysis of positioning error only, and not used as the guiding mark. Besides, the standard deviation of the estimated velocity error between the GPS and the vision system was approximately 0.1 m/s in both *x* and *y* directions. The close agreement suggested that the vision-based aid is competitive with the conventional GPS-based in terms of velocity estimation. 

Figure 11a shows the in-plane route during the vision-based landing, where the target spot $P_{0}$ (black cross) was set at the origin, corresponding to the center of camera view (b). Eventually, the UAV landed at the location marked as a green dot ($P_{\mathrm{gps}}$) and a blue dot ($P_{\mathrm{vision}}$) according to the reading of GPS and visual sensor, respectively. In order to authenticate the landing position (Supplementary Figure S1a), we verified the landing accuracy by the template matching method, as shown in Figure 11b. In terms of overview, the detected location $P_{g}$ was treated as the ground truth, and the corresponding coordinate was also marked by a red cross in Figure 11a. As can be seen, the proposed vision-based system can precisely guide the UAV landing with the in-plane positioning error of approximately 0.1 m.

**Figure 12:**
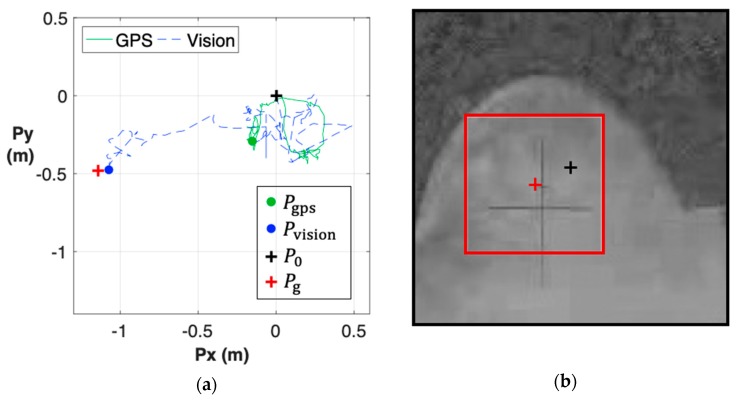
Mode 2 (GPS-based landing): (**a**) After descending a certain height, the estimated position of vision system (blue dot) was more accurate than the GPS (green dot); (**b**) The corresponding template matching result indicated that the UAV’s position was approximately 1.2 m away from the designated landing spot.

For comparison purposes, we conducted the landing process using the measurements from GPS instead of the proposed vision system. Like the previous case, the flight control system set the designated landing spot at the center of the camera view when the landing control starts (black cross in Figure 12). When the UAV descended by 7 meters, there was a difference in position estimation between these two systems. Similarly, we applied the template matching method to authenticate the position (Supplementary Figure S1b), and the finding result was marked by a red cross ($P_{g}$). As shown in the figures, the GPS-based landing resulted in an approximate 1.2-m of position error, higher than the one by the vision-based landing. Meanwhile, the position estimate of the vision system was closer to the ground truth. To demonstrate that no specific marker is required for the proposed system, we performed additional experiments with different initial conditions (Supplementary Figure S2) and obtained similar results. The landing performance with vision-aided (mode 1) and GPS-aided (mode 2) modes are summarized in Table 1. Regardless of whether GPS has a biased error or not, the vision-aided scheme can effectively avoid the possible sensing error, and lead to an accurate landing performance.

## 4. Conclusions

In this paper, we proposed a hybrid vision-based framework to maneuver UAV landing without prior knowledge about the landing spot. Unlike the past experiences in which a well-defined marker is required for motion detection, we introduced a hybrid scheme to select the operating images and algorithms based on the estimated motion; thus, meeting the need to land in an unvisited area with no target recognition required. In this work, the GFA and PC methods were combined to compromise both advantages of dense and sparse optical flow in terms of accuracy and robustness, and a multi-scale strategy is proposed to tackle the field-of-view problem during UAV descent. The experimental results indicated that the UAV is able to stabilize its velocity and position in the absence of GPS signals. Finally, with the vision-based motion estimation system, we successfully carried out the UAV landing more precisely than is achieved by using a GPS in an outdoor environment

The proposed vision-aided system not only accomplished the landing mission with a superior performance to the conventional GPS but had full potential to incorporate fast-growing imaging and learning procedures. For an autonomous UAV to land safely at an unvisited airfield, a survey to assess the planarity of the landing surface must be considered prior to the final approach phase. Therefore, based on this work, we will extend the machine vision upon landing site selection and apply self-supervised learning to autonomously locate a safe landing spot in an unsurveyed environment. 

## Figures and Tables

**Table 1:** Comparison of positioning performance. (unit: m).

Landing Mode	Landing Target	Final Position	GPS	Vision System
	$P_{0}$(+)	$P_{g}$(+)	$∆P_{\mathrm{gps}}$(● - +)	$∆P_{\mathrm{vision}}$(● - +)
Mode 1	(0, 0)	(0.11, 0.07)	(0, −0.13)	(−0.08, −0.04)
Mode 1	(0, 0)	(−0.07, 0.40)	(0.91, 0.93)	(−0.02, −0.30)
Mode 1	(0, 0)	(−0.45, −0.05)	(0.05, 0.58)	(0.47, 0.05)
Mode 2	(0, 0)	(−1.14, −0.48)	(0.99, 0.18)	(0.06. 0.01)

## Supplementary Materials

The following are available online at https://www.mdpi.com/1424-8220/19/6/1380/s1, Figure S1: The images used for template matching. Figure S2: Mode 1 (vision-based landing): the results associated with different initial conditions.
