# Supplementary material for: Motion Estimation by Hybrid Optical Flow Technology for UAV Landing in an Unvisited Area
Source: Sensors (Basel). 2019 Mar 20;19(6):1380. doi: 10.3390/s19061380 (PMC6471152; doi:10.3390/s19061380)
Supplement: Supplementary file 1 [file sensors-19-01380-s001.pdf]

sensors
MDPI

Supplementary Materials

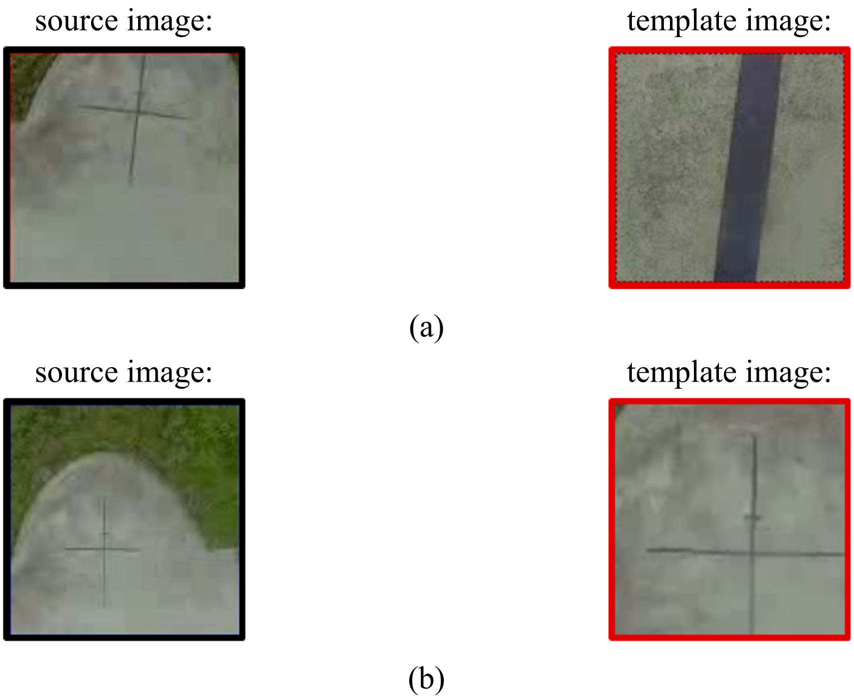

Figure S1. The images used for template matching: (**a**) vision-based landing; (**b**) GPS-based landing.

To demonstrate that no specific landing marker is required for the proposed visual motion estimation, we conducted a number of experiments with different initial conditions. The designated landing spot and the starting height were randomly selected by the pilot. These results indicated that the vision system is able to overcome the GPS drift problem and improve the landing accuracy.

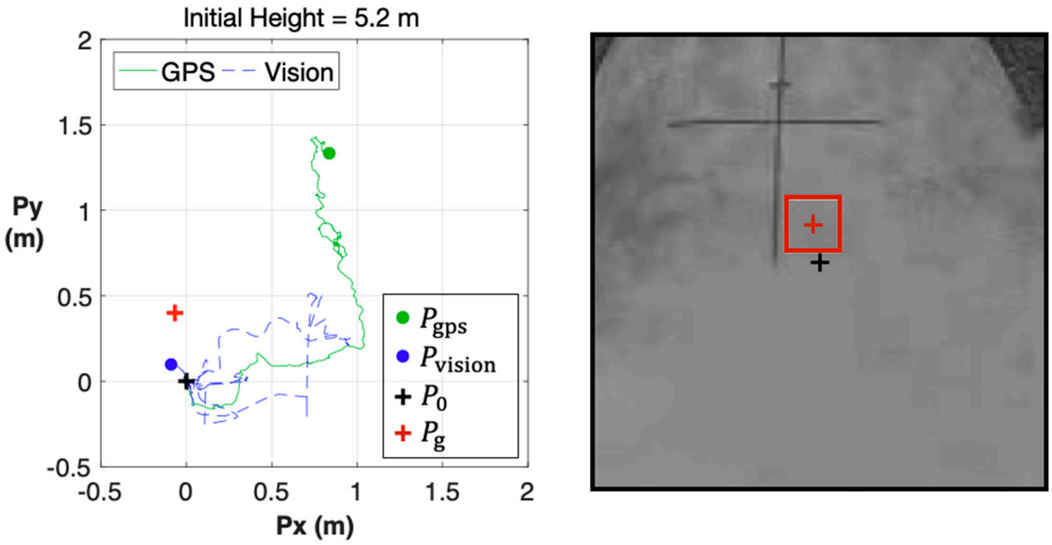

(a)

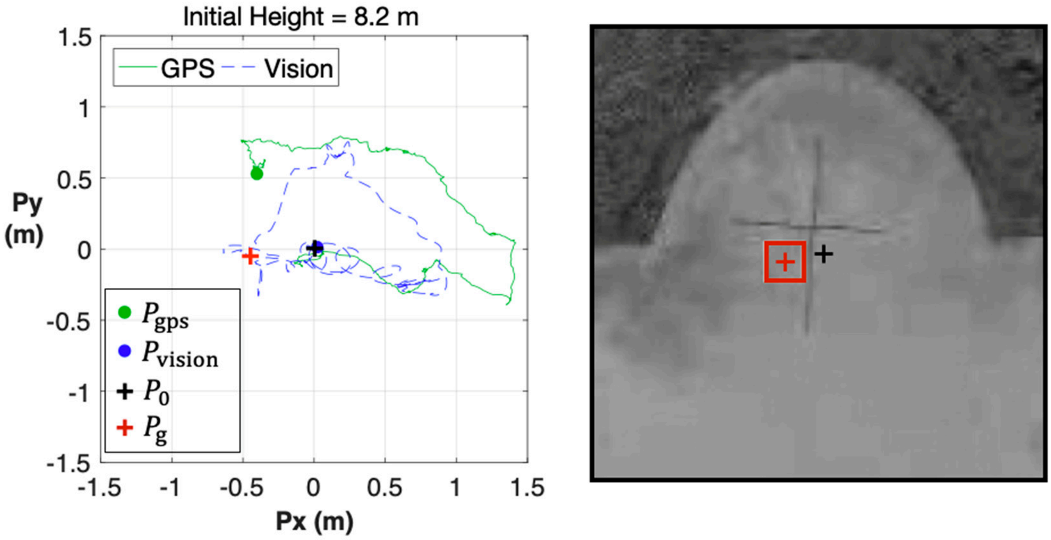

(b)

Figure S2. Mode 1 (vision-based landing): the results associated with different initial conditions.
